# The yeast gene ECM9 regulates cell wall maintenance and cell division in stress conditions

**DOI:** 10.17912/micropub.biology.001313

**Published:** 2024-10-01

**Authors:** Prevena Ramakrishnan, Jill Keeney

**Affiliations:** 1 Biology, Juniata College, Huntingdon, Pennsylvania, United States

## Abstract

*Saccharomyces cerevisiae* , Baker's yeast, is a well-studied model eukaryotic organism. Much of our knowledge about eukaryotic cell function comes from yeast studies, though nearly 10% of yeast genes remain uncharacterized. This study focuses on YKR004C, a verified gene of unknown function named *ECM9* , predicted to be involved in cell division and cell wall maintenance or composition based on previous studies. We investigated the sensitivity in stress conditions of an *ECM9* deletion strain, compared to wild-type, to cell wall integrity. These results suggest that *ECM9* is involved in cell wall maintenance and the regulatory pathway determining cell division readiness under stress.

**Figure 1. ECM9 deletion displays different phenotypes in stress conditions:**
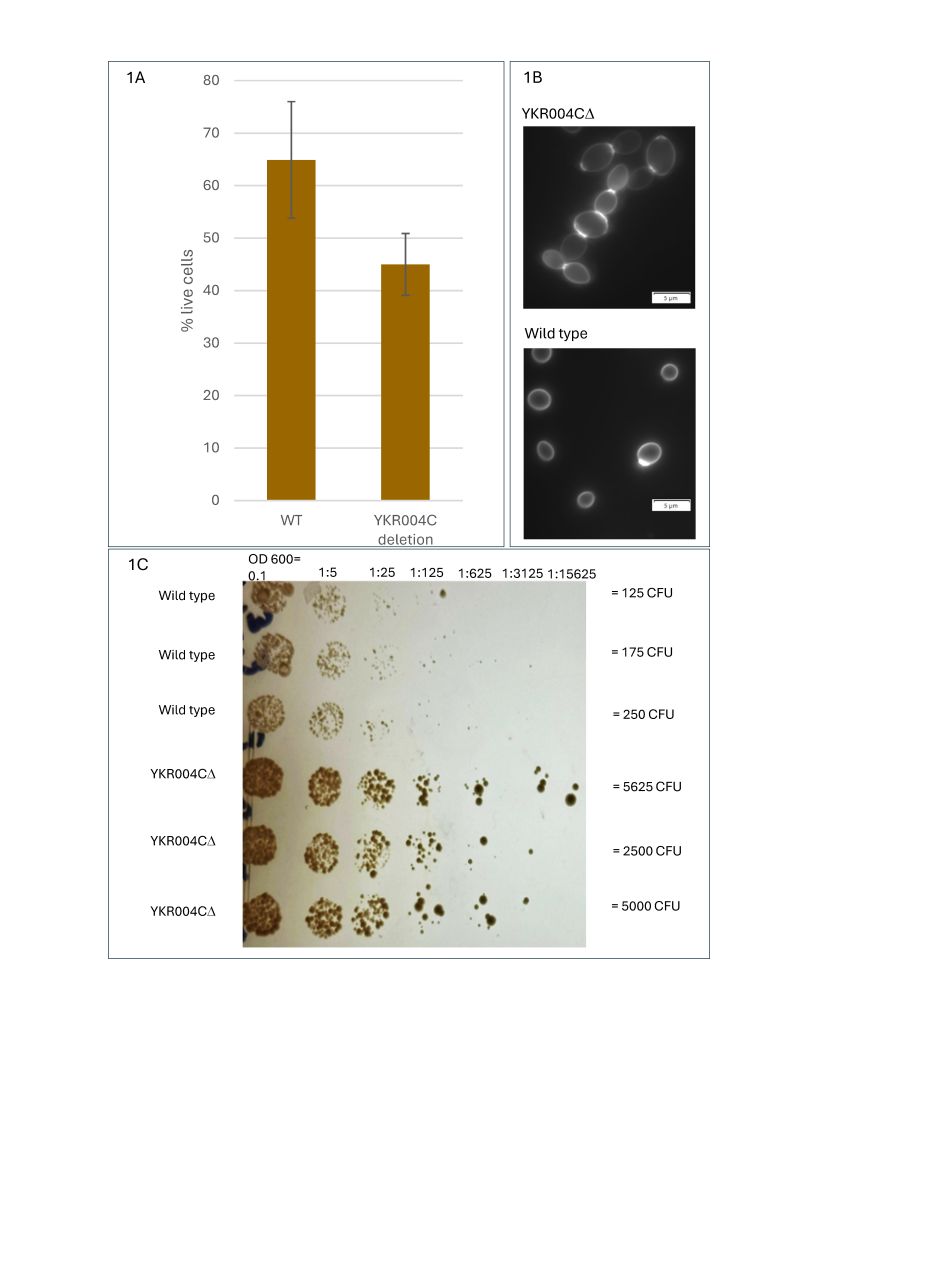
**1A** : Osmotic shock test to measure cell wall fragility between wild-type (WT) and *ecm9* deletion strains. Cells were grown overnight in YPD with 0.5 M sorbitol, suspended in water for 10 minutes, stained with 0.1% (w/v) methylene blue solution and cell viability determined by cell counts. The bar graph shows the average percent live cells of three trials; the error bars indicate the standard deviation. **1B:** *ecm9* deletion and wild-type strains grown in YPD medium with 0.5M sorbitol, stained with calcofluor white stain and viewed at 100x under DAPI setting. **1C** : Spot assay of wild-type and *ecm9* deletion strains. Cells were grown to log phase, diluted to an OD _600_ =0.1, serially diluted, and 2 uL of the original and each dilution spotted onto YPD plates containing calcofluor white at a final concentration of 50 μg/mL. Cell dilutions are given above each column; the top three rows are replicates of wild-type and the bottom three rows of the *ecm9* (YKR004C) deletion strain. Calculated CFUs for the OD _600_ =0.1 cultures are indicated.

## Description

YKR004C or *ECM9* , a gene in the model yeast *Saccharomyces cerevisiae* , produces a verified, non-essential protein of unknown function, containing an intron. The three controlled Gene Ontology (GO) categories- molecular function, biological process and cellular component-are unknown (Ashburner et al., 2000; Gene Ontology Consortium et al., 2023; Wong et al., 2023) . *ECM9* , ExtraCellular Mutant 9, was named from a screen of the yeast deletion library for strains with altered sensitivity to calcofluor white (CFW), indicating a possible role in cell wall assembly (Lussier et al., 1997) . Additionally, a previous study by Dungrawala et al (Dungrawala et al., 2012) showed that deletion of ECM9 resulted in larger virgin daughter cells compared to the wild-type, suggesting a role in cell size control. With this limited information we conducted experiments to further investigate the function of *ECM9.*

The yeast cell wall is composed of chitin, glucan (β-1,3 and β-1,6), and mannoproteins (Kollár et al., 1997; Orlean, 2012). These components are not static; instead, they are actively managed and highly controlled by the cell. Their levels and proportions can change in response to two main factors- stress conditions that the cell experiences and different stages of the cell's life cycle (Gomar-Alba et al., 2015) . Sorbitol-induced osmotic stress reduces chitin biosynthesis, as evidenced by lower expression of chitin biosynthesis genes (Gomar-Alba et al., 2015) . Despite chitin comprising only 2-3% of the cell wall, primarily in the bud scars, its regulation is crucial, and changes in its levels affect sensitivity to CFW (Gomar-Alba et al., 2015; Perrine-Walker & Payne, 2021) . Specifically, decreased chitin content, as seen with sorbitol, increases resistance to CFW, while increased chitin levels enhance sensitivity (Popolo et al., 1997; Ram et al., 1998; Shaw et al., 1991) . This dynamic regulation allows the yeast to adapt its cell wall structure to best suit its current environment and growth phase.

Since *ECM9* has been implicated in cell wall function, we did an osmotic shock test to determine if there is any difference in cell wall fragility between wild-type (WT) and *ecm9* deletion strains (Hartland et al., 1994) . Strains were grown overnight in rich (YPD) media supplemented with 0.5M Sorbitol to induce an osmotic response and then suspended in a hypotonic medium. After 10 minutes, cells were stained with 0.1%(w/v) methylene blue solution, and a viability count was performed by counting clear and blue cells, as dead cells cannot exclude the methylene blue. An average of three trials showed 64.9% of wild-type cells were alive compared to only 45.0% of *ecm9* deletion cells. ( Figure 1A ; 2-sample, 2-tail t test; p-value = 0.05). This result suggests that the *ecm9* mutant strain has reduced viability in osmotic stress, supporting the hypothesis that *ECM9* may play a role in cell wall maintenance or composition.

It has been shown that deletion of *ECM9* results in statistically larger virgin daughter cells compared to wild-type, but no change in budding index, a key observation indicating that *ECM9* plays a role in regulating cell size (Dungrawala et al., 2012) . The critical cell size is the threshold size that cells must reach before committing to cell division at the G1/S transition, often referred to as the restriction point, or START. This is a crucial regulatory checkpoint in the cell cycle, ensuring that cells only divide once they have reached a sufficient size to support the subsequent daughter cells. The *ECM9* mutant cells reach this critical size at a larger volume than wild-type cells (Dungrawala et al., 2012) . By requiring cells to grow larger before division, the absence of *ECM9* may impact overall cell cycle timing and efficiency.

To directly observe the cell wall morphology and cell size, we did calcofluor white staining of wild-type and *ecm9* deletion strains grown in YPD medium and YPD medium with 0.5 M sorbitol (Perrine-Walker & Payne, 2021) . Mutant strains grown in YPD + 0.5M sorbitol media are notably larger and show an unusual budding style ( Figure 1B ). Contrary to wild-type yeast, these mutant cells did not separate during cell division but rather formed pseudohyphae. The pseudohyphae do not display invasive agar growth, as cells grown on sorbitol containing medium were easily washed away.

In addition to its use as a fungal stain, calcofluor white (CFW) is a perturbing chemical agent that binds to nascent chitin chains which prevents microfibril assembly and interferes with cell wall organization (Elorza et al., 1983; Sanz et al., 2018) . Thus, we did a spot assay, growing wild type and *ecm9* mutant cells on 50 ug/mL CFW. ( Figure 1C ). The *ecm9* deletion strain shows significant resistance to calcofluor white compared to wild-type. The average total CFUs (colony forming units) in the undiluted culture was 183 for wild type and 4375 for the *ecm9* deletion strain (2-sample, 2-tail t test; p-value=0.01). This is contradictory to the screen for extracellular mutants in which an *ecm9* transposon insertion mutant was found to be sensitive to CFW (Lussier et al., 1997) . A key difference is the background strain used in the original screen, AWM3CD630, and the S288C-derived *ecm9* deletion strain used in this study.

Our results of pseudohyphae formation in an *ecm9* deletion strain under osmotic stress ( Figure 1B ) imply a delay in G2/M progression, which causes cells to grow longer because they spend more time in a phase where growth is directed toward the cell’s apex (tip). (Song Q et al., 2012). This suggests that *ecm9* deletion cells may be unable to properly regulate the timing of bud initiation and separation of the cell cycle under stress conditions. We also observed a larger cell-size. Our findings implicate a cell cycle defect at G2/M in addition to the G1/S restriction point defect previously described for *ECM9* (Dungrawala et al., 2012) .

At present, the cellular localization of Ecm9p is unknown, with localization algorithms placing the protein in the cytoplasm by default. Annotated physical interactors of Ecm9p include a handful of genes that broadly regulate mRNA (Wong et al., 2023) . The decreased viability of *ecm9* deletion strains under osmotic stress and the increased resistance to CFW implies that *ECM9* , in addition to cell cycle regulation, is also involved in maintaining cell wall structure and function, indicating pleiotropy. Without *ECM9* , cells may be less capable of withstanding changes in osmotic pressure, leading to increased cell damage or death, perhaps due to changes in chitin content. The role of *ECM9* in cell wall composition and response to osmotic stress aligns with its potential involvement in broader cellular stress response pathways, including those regulating pseudohyphal growth.

## Methods

**Yeast growth.** Yeast were grown at 30°C in YPD (US Biological Life Sciences, Salem, MA) medium in liquid cultures or on solid medium with 2% agar. *Saccharomyces* *cerevisiae* strains used in this study are listed in Table 1. The *ecm9* deletion was purchased from Horizon Discovery (Lafayette, CO) and confirmed using colony PCR (Lõoke et al., 2011). For colony PCR, primers (IDT Technology) were: forward primer upstream of ECM9 coding sequence; 5’-AGTCATGAAACCATTGCGCT-3’; and the reverse primer to the *kanmx* cassette, 3’-ATCACTCGCATCAACCAAACC-5’. The PCR reaction contained 2x Taq mix (New England Biolabs ®, Ipswich, MA), 0.8 µM primer final concentrations and 1 uL of genomic DNA. PCR conditions were: 95°C for 30 seconds, 52°C for 30 seconds, 68°C for 3 min, 35 cycles, followed by a finishing cycle of 68°C for 5 minutes.

**Osmotic test shock** was done as described in Hartland et al., 1994. Cells were grown overnight in YPD with 0.5 M sorbitol (Sigma-Aldrich ®, St. Louis, MO) 240 μL of each yeast culture was pelleted and media was aspirated. 225 μL of water was added and vortexed, and the cells were let to sit in water for 20 minutes. Yeast cells were stained with 10 μL of methylene blue (0.1%, w/v), loaded into a hemocytometer and at least 100 cells of each sample assessed for viability.

**Calcofluor staining.** Log phase yeast cells grown in YPD and YPD + 0.5M Sorbitol were washed with water twice before staining with 10 μL of 1g/L calcofluor white (Sigma-Aldrich®, St. Louis, MO). Cells were viewed using a fluorescence microscope (Olympus IX81, cellSens Dimension Software) with DAPI fluor setting at a 100X magnification.

**Spot assay** was done as described (Petropavlovskiy et al., 2020) . Wild type and deletion strains were grown to log phase, diluted to an OD600 of 0.1 and then serially diluted 1:5; 2 μL of cells of each dilution were spotted to rich (YPD) media and 50 μg/ml calcofluor white media.

**Table:** 

**Name**	**Genotype**	**Available from**	**Reference**
BY4742 (wild type)	*MATα his3Δ1 leu2Δ0 lys2Δ0 ura3Δ0*	ATCC	Brachmann et al., 1998
*ecm9 Δ*	*MATα his3Δ1 leu2Δ0 lys2Δ0 ura3Δ0 YKR004CΔ::kanmx4*	Horizon Discovery	Giaever et al., 2002
